# The theory of discovering rare variants via DNA sequencing

**DOI:** 10.1186/1471-2164-10-485

**Published:** 2009-10-20

**Authors:** Michael C Wendl, Richard K Wilson

**Affiliations:** 1 The Genome Center and Department of Genetics, Washington University, St. Louis MO 63108, USA

## Abstract

**Background:**

Rare population variants are known to have important biomedical implications, but their systematic discovery has only recently been enabled by advances in DNA sequencing. The design process of a discovery project remains formidable, being limited to *ad hoc *mixtures of extensive computer simulation and pilot sequencing. Here, the task is examined from a general mathematical perspective.

**Results:**

We pose and solve the population sequencing design problem and subsequently apply standard optimization techniques that maximize the discovery probability. Emphasis is placed on cases whose discovery thresholds place them within reach of current technologies. We find that parameter values characteristic of rare-variant projects lead to a general, yet remarkably simple set of optimization rules. Specifically, optimal processing occurs at constant values of the per-sample redundancy, refuting current notions that sample size should be selected outright. Optimal project-wide redundancy and sample size are then shown to be inversely proportional to the desired variant frequency. A second family of constants governs these relationships, permitting one to immediately establish the most efficient settings for a given set of discovery conditions. Our results largely concur with the empirical design of the Thousand Genomes Project, though they furnish some additional refinement.

**Conclusion:**

The optimization principles reported here dramatically simplify the design process and should be broadly useful as rare-variant projects become both more important and routine in the future.

## Background

Technological developments continue to dramatically expand the enterprise of DNA sequencing. In particular, the emergence of so-called "next-generation" instruments (NGIs) is opening a new chapter of genomic research [1]. If we characterize sequencing economy by the ratio of project speed to total project cost, NGIs are orders of magnitude superior to their traditional Sanger-based predecessors. Indeed, they are the first systems to demonstrate the economic feasibility of sequencing individual genomes on a large scale [2].

Future efforts will undoubtedly use NGIs to address issues in medical sequencing and personal genomics [3], but these instruments are also poised for major contributions at the population level [4,5]. For example, the Thousand Genomes Project (TGP) is focusing on comprehensive identification of variants in the human population through cohort-level whole-genome sequencing using NGIs [6,7]. One of its main goals is to discover and characterize rare single nucleotide alleles, basically those present at minor allele frequencies around 1% or less. This region was not accessible to the earlier HapMap Project [8]. Rarer instances are obviously much more difficult to find and necessitate gathering enormously larger amounts of data. Such demands will obviously extend to any future such projects one might envision, including those for model organisms, agriculturally important species, cancer genomes, infectious agents, etc.

The success of such variation projects depends upon adequately understanding the relevant process engineering issues and subsequently crafting a suitable project design. One concern in traditional single-genome sequencing is the so-called "stopping problem" [9-11]], which is the proposition of estimating what redundancy will suffice for a desired level of genomic coverage. Variation projects similarly require specification of a total, project-wide redundancy, *R*. Yet, because they necessarily involve multiple genomes, an essentially new design question also emerges. That is, how does one optimize the number of samples, *σ*, versus the redundancy allotted per sample, *ρ*, such that the probability of finding a rare variant, *P*_*v*_, is maximized? The existence of such optima is intuitively clear. Heavily sequencing only a few samples will tend to miss a variant because it is unlikely to be present in the original sample set. Conversely, light sequencing of too many samples may overlook the variant by virtue of insufficient coverage for any samples actually harboring it. Somewhere between these extremes lie optimum combinations of parameters.

At present, this issue can only be addressed in *ad hoc*, fairly inefficient ways. For example, the TGP conducted both painstaking computer simulations and pilot sequencing phases involving hundreds of genomes to aid in designing the full-scale project [6,7]. While certainly informative, even such seemingly extensive data may not, by themselves, give a complete picture of optimization because combinations of the many underlying variables (Table 1) lead to an enormous solution space. We comment further on this aspect below. Existing theory is also ineffective because sequence coverage has not yet been considered [12].

**Table 1:** Variables in a Multi-Genome Variant Detection Project

**variable**^**†**^	meaning
*P*_*v*_	probability of finding a rare variant
*P*_*v*, *min*_	minimum acceptable value of *P*_*v *_for a project
*ρ*	haploid per-sample sequence redundancy
*R*	total, project-wide redundancy
*ϕ*	frequency of variant in population
*σ*	number of samples sequenced in project
*τ*	minimum read coverings for detection
*N*	minimum variant observations to declare discovery

^†^Some variables are modified with a "star" superscript to denote optima, for example σ* is the optimum sample size for a project, ρ* the optimum per-sample redundancy, and  the discovery probability under optimal conditions.

Here, we examine optimization from a more focused mathematical perspective. Our treatment accounts for sequence errors via the proxy of a variable read covering count [3,13], but it omits secondary, project-specific details like software idiosyncrasies [14], instrument-specific biases [15], and alignment issues [16]. The solution leads to a set of general, though unexpectedly simple optimization principles, which correct some earlier speculation [17] and are useful as first approximations for actual projects. Because these rules appreciably narrow the solution space, they also offer good starting points for even more targeted numerical and empirical searches that might account for secondary effects, if such are deemed necessary.

## Results

The term "rare variant" is routinely taken to mean a rare allele, although it can also mean a rare SNP genotype. Take *ϕ *to be the variant frequency, i.e. the minor allele frequency or the rare homozygous genotype frequency, as appropriate. We assume the TGP convention whereby samples are sequenced separately to uniform depths [6,7], instead of being pooled first. The general theory then encompasses the multiple-genome population sequencing problem and its subsequent design optimization.

### Analytical Characterization of Discovery in Multiple Genomes

**Theorem 1 (Allele Variants)**. Let *D*_*A *_be the event that a rare allele is detected, i.e. found by the investigator in a sequenced diploid genome sample. Its probability is(1)

where(2)

is the coverage probability of spanning the allele's genomic position on a chromosome with at least *τ *sequence reads. Let *σ *independent, randomly-selected samples each be sequenced uniformly to haploid depth *ρ*. Then, if *K *is the random variable representing the number of samples the variant is found in and if *N *is the minimum number of observations necessary to declare the variant as being "discovered", the discovery event is defined as *K *≥ *N *and its probability is(3)

**Theorem 2 (Genotype Variants)**. The probability of *D*_*G*_, the event that a rare genotype is detected in a sample, is(4)

and its discovery probability is again given by Eq. 3, except where *D*_*G*_, replaces *D*_*A*_.

### Statement of the Optimization Problem

Variant discovery is a constrained optimization problem [18], which can be stated as follows. Given the biological parameter *ϕ *and project-specific design parameters *R*, *σ*, *τ*, *P*_*v*, *min*_, and *N*, maximize the objective function *P*_*v*_, subject to both the equality constraint(5)

and to the auxiliary constraint(6)

In practical terms, we want to most efficiently discover a variant at the lowest possible cost, as represented by *R*.

Although the problem is framed in terms of finding a single variant, actual projects are apt to be specified according to discovering a certain average number of rare variants. These scenarios are equivalent, as Eq. 6 also quantifies the expected fraction of variants that will be found in the project. For example, *P*_*v*, *min *_= 0.95 indicates finding 95%, on average, of the variants occurring at some value of *ϕ*.

### Optimizing for Single and Double Variant Observations

Leaving aside the optimization of *ρ *versus *σ *for a moment, the least obvious of the project-specific parameters to specify is arguably *N*. Higher values may exceed the actual number of instances in the sample set, resulting in *a priori *failure of the project. We will therefore concentrate on the experimentally relevant special cases *N *= 1 and *N *= 2. The former is clearly a minimum requirement, while the latter serves to better discern between a rare population variant and a SNP that is unique to an individual sample (a "private SNP").

Because we have an explicitly-defined equality constraint in the form of Eq. 5, the number of design variables can be reduced by one [18]. Specifically, substituting *ρ *= *R*/*σ *into Eq. 2 allows us to write a constrained form of the coverage probability, which in turn furnishes constrained expressions for the probabilities of events *D*_*A *_and *D*_*G*_. It is expedient at this point to switch from the event-based notation of probability used up until now to the Eulerian (functional) convention for the calculus-based aspect of optimization. Specifically, let *f*_*τ*, *i*_with *i *∈ {*A, G*} represent the now-constrained probabilities of *D*_*A *_and *D*_*G*_. (A detailed explanation of the switch in notation appears in "Mathematical Preliminaries".) We now state the following important optimization conditions.

**Theorem 3 (Optimal Conditions)**. The optimum number of samples in a multiple-genome variation project for *N *= 1 is governed by the differential equation(7)

and for *N *= 2 by the differential equation(8)

In particular, the roots of these equations in *σ *indicate maxima in *P*_*v *_for rare alleles and genotypes. Each setting of the independent variables has one such optimum, σ*, which is necessarily a global optimum.

## Discussion

Finding rare variants is clearly an important aspect of both population and medical genetics [19]. The discovery process was not feasible before the advent of NGIs, but is now being actively prototyped through efforts like the TGP [6,7] and will likely become more routine in the future. This eventuality motivates examination of the problem from a general perspective, similar in spirit to theoretical treatments of single genomes [20]. The following sections report on both some of the broad trends across the design variable spectrum, as well as optimal conditions for the important special cases of *N *= 1 and *N *= 2.

### General Trends

**Figure 1:**
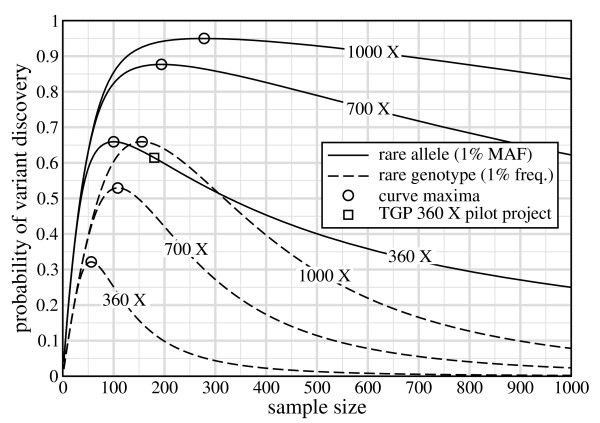
**Probability of discovering variants at *ϕ *= 1% as a function of sample size for *N *= 1 and *τ *= 2**. The single square datum represents the TGP pilot project at *R *= 360×. Circles indicate maxima for each curve.

Fig. 1 shows *P*_*v *_versus *σ *for variants appearing at 1% frequency for thresholds of *N *= 1 and *τ *= 2. The latter appears to have emerged as the *de facto *choice to better control for sequencing errors [3,13]. Aside from the expected trend that performance improves as more data are gathered, the curves show two notable properties. First, σ*, the sample size at which the maximum *P*_*v *_occurs, increases with the project redundancy. This dependence means that a project cannot generally be optimized by selecting *σ *in advance of other factors. Put another way, outright specification of *σ *almost certainly assures that the discovery process will not be optimal. We expand further upon this point below.

Fig. 1 also shows that curves are not symmetric with respect to σ*. The rate of drop-off of *P*_*v *_for a given project-wide redundancy is much more severe for *σ *<σ*, implying that it is better to err in sequencing too many samples rather than too few. It is interesting to examine one of the TGP sequencing pilot phases in this context, which specifies 2× data collection for each of *σ *= 180 samples [6,7]. Here, *R *= 2·180 = 360, which is one of the curves plotted in Fig. 1. Using the above thresholds, this design yields *P*_*v *_≈ 61%, whereas the optimal configuration returns *P*_*v *_≈ 66% for only about 100 samples. Despite using almost twice as many samples as is optimal, this design remains relatively good, precisely because of the non-symmetric behavior.

### Constant Sample-Size Designs and the Stalling Effect

**Figure 2:**
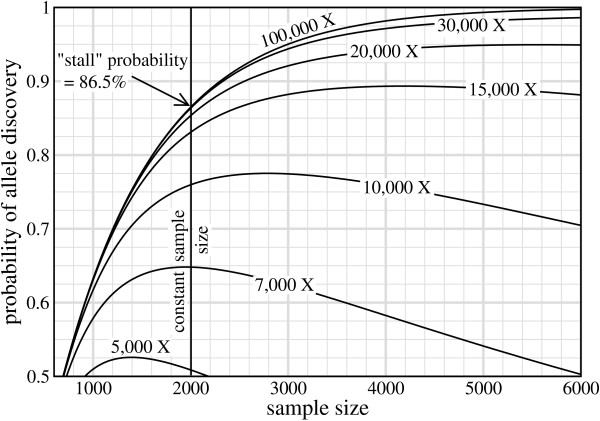
**Gibbs' scenario **[17]**of using a fixed 2,000 unit sample size to discover extremely rare alleles, *ϕ *= 0.0005, under *N *= 1 and *τ *= 2**. This hypothetical project is plotted for 5,000 ≤ *R *≤ 100,000 and shows the conspicuous "stalling" effect that occurs under increasingly non-optimal conditions.

The above discussion suggests that investigators should consider abandoning the idea of choosing *σ *outright. An earlier projection offers an interesting case study to further illustrate this point. Gibbs [17] postulated that *σ *= 2,000 samples would be a good way of discovering extremely rare variants occurring at 0.05%. (This number may simply have been an expeditious choice, as further details were not specified, nor was there any description of how this prediction was made.) Fig. 2 shows the implications of such a *σ*-based design. As *R *increases, σ* marches to the right on the abscissa, eventually passing through the pre-selected *σ *= 2,000 at around *R *= 7,000. It continues rightward, leaving our fixed sample datum in the left-side wake of the optimum (*σ *<σ*, as mentioned above), where the associated probability is now heavily penalized. In fact, the probability stalls at a value of roughly *P*_*v *_≈ 0.85, regardless of the amount of additional data poured into the project.

Although this stalling effect may initially seem counter-intuitive, its explanation is quite straightforward. If we hold *σ *fixed while letting *R *increase without bounds, then *ρ *also grows without bounds (Eq. 5). In the limit, each sample will be perfectly sequenced, i.e. *P(C) *→ 1 in Eq. 2. Discovery is then simply a function of whether or not the variant is present in the original sample set. If so, it is absolutely certain to be discovered. The corresponding probabilities are then simple special cases of the model in Thms. 1 and 2. For example, for *N *= 1 observation of a rare allele we find(9)

which is asymptotically identical to what is obtained if coverage is not considered at all [5]. The basic problem associated with constant sample-size designs is immediately apparent in this equation. Given small *ϕ*, the exponential term decays very slowly and can only be compensated for by increasing *σ*. The challenge, of course, is to do this such that *P*_*v *_attains a maximum.

### Remarks on Optimization Methods

We commented above that empirical prototyping and numerical simulation are unlikely to give complete insights to the general optimization problem because of the size of the solution space. Consider that the relationship between two parameters requires only a single curve on an X-Y plot, three parameters require a family of curves on one plot, four a textbook of family-type plots, and so forth. Richard Bellman, who developed the optimization technique of dynamic programming, called this phenomenon the "curse of dimensionality". Table 1 shows that we have 8 variables in our particular problem, however, even this is somewhat misleading because it does not consider the probabilistic nature of the problem. That is, *P*_*v *_can only be established as an expected value through a sufficient number of repeated trials for each particular combination of the independent variables. This is the basic tactic used in simulation.

The population model in Thms. 1 and 2 improves matters considerably, furnishing *P*_*v *_explicitly in terms of (*τ*, *R*, *σ*, *ϕ*, *N*). One could march through every combination of these variables, evaluating *P*_*v *_for each, and log maxima that attain given levels of *P*_*v*, *min*_. Though this approach would be enormously more efficient than naïve brute-force simulation, the calculations needed to adequately survey the floating-point "continuum" of the real-valued variables remain basically infeasible. Consequently, we still might not expect to discern any latent general laws.

### The Weak Optimization Problem

We resort instead to Thm. 3, whose roots for *N *= 1 and *N *= 2 represent optimal sample sizes, σ*. Let us first describe some unexpected properties found among the independent variables. These are important in that they furnish a direct solution to what might be called the *weak *optimization problem. This is the proposition that relaxes the condition defined by *P*_*v*, *min*_. In effect, weak optimization provides the optimal probability, , subject to a pre-determined R rather than a given *P*_*v*, *min *_> 0.

**Figure 3:**
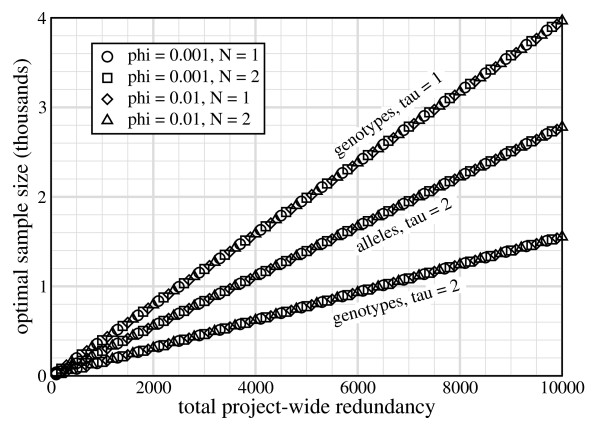
**Optimal sample size versus project-wide redundancy for parameters representative of rare-variant projects**.

Fig. 3 shows σ* versus *R *for representative parameter settings. Data collapse onto curves according to variant type. In each case, σ* = σ*(*R*, *τ*) and σ* ∝ *R*. These observations, coupled with σ* = *R*/ρ* from Eq. 5 then imply σ*(*R*, *τ*) = *R*/ρ*(*τ*). In other words, ρ* is only a function of *τ *(Table 2). This is quite a significant finding because it immediately establishes the best sample redundancy for a project. In essence, this observation indicates that optimizable designs for rare variants are based on constant values of *ρ *rather than constant values of *σ *[17].

**Table 2:** Constants Associated with Optimum Designs

rare variant	*τ*	ρ*(*τ*)		
genotype	1	2.5	0.512	2.5
genotype	2	6.4	0.690	6.4
allele	2	3.6	0.537	1.8

allele	1	special case, see Eq. 10		

^†^See Eqs. 14, 15, 16

**Figure 4:**
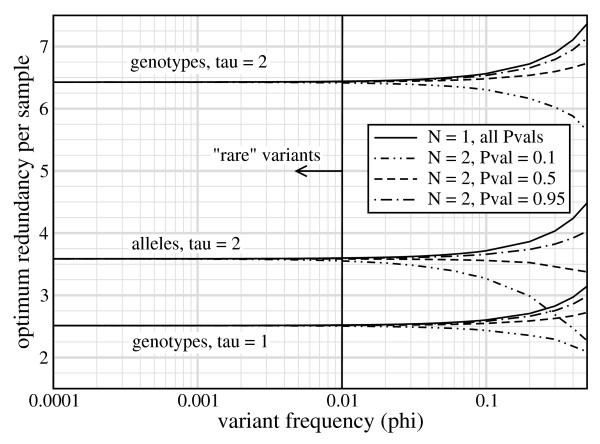
Optimum redundancy per sample, ρ*, is essentially constant for each value of *τ *within the conventional range of *ϕ *≤ 1% for rare variants.

We emphasize that the numbers in Table 2 are not based on first-principles and are not strictly encoded in the governing equations. Rather, they are fortuitous empiricisms, restricted to the parameter values that characterize rare-variant projects. Fig. 4 demonstrates that, while ρ* does indeed only depend upon *τ *up to allele frequencies of about 1%, it becomes a more complicated function of additional variables for higher frequencies.

### Remarks on the Special Case of *τ *= 1 for Rare Alleles

The case of *τ *= 1 is conspicuously absent for rare alleles in Figs. 3 and 4 because its optimum sample size is not finite. Unlike the other cases, *P*_*v *_approaches its maximum as *σ *→ ∞, for example(10)

Here, we have the seemingly contradictory implication that we should spread a finite amount of sequence resources over the largest number of samples, each of which will then have a vanishingly small *ρ*. Mathematically speaking, the rate by which the per-sample *f*_1, *A *_decreases precisely offsets the favorable rate of increasing sample size, whereby *P*_*v *_does not asymptotically vanish. However, there will usually be good secondary reasons for limiting *σ *in practice, e.g. cost of sample procurement. Moreover, conditions approach the limiting value rather quickly, for example setting *ρ *= *R*/*σ *≤ 0.1 brings *P*_*v *_very close to the expression in Eq. 10. *R *is the main factor governing discovery under these conditions and its value can be calculated for a desired *P*_*v *_by simply inverting Eq. 10.

### Optimal Designs for Single and Double Variant Observations

**Figure 5:**
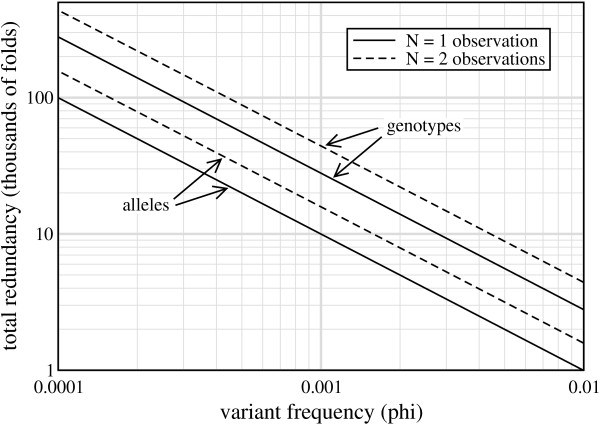
Log-log plot of optimum designs, R* vs. *ϕ*, for discovering rare genotypes and alleles at probabilities of 95% when requiring at least two read coverings (*τ *= 2).

The weak solution specifies constants of ρ* (Table 2), which simultaneously imply σ* for any choice of *R*. These properties subsequently fix certain relationships within the general problem, so that optimization for a desired *P*_*v*, *min *_in Eq. 6 reduces to the task of solving directly for *ϕ *(see Methods). Fig. 5 shows the resulting optimal designs for *τ *= 2, a setting characteristic of recent projects [3,13]. Results are plotted for *P*_*v*, *min *_= 95%, the same threshold set by the TGP. All curves show a special kind of log-log relationship between *ϕ *and R* in which the slope is -1. In other words, optimal designs can be expressed as a family of log-log curves having the form ϕ R* = *C*(*N*, *τ*, *P*_*v*, *min*_), where *C *is a so-called *optimization coefficient *for each combination of the variables. Of course, knowing *C *immediately enables one to compute R* and subsequently σ* = R*/ρ* for a desired *ϕ*, which is of enormous practical value for project design. Table 3 shows *C *for the configurations having well-defined optimum redundancies, although we note that Eq. 10 also follows this form, having *C *= 3.0. R* is indicative of the total resources a project requires, so *C *is also useful in comparing relative costs. For example, requiring two observations of a rare allele instead of just one would only be, somewhat counter-intuitively, about 60% more expensive if both schemes were to be done optimally.

**Table 3:** Optimization Coefficients for 95% Discovery Probability

variant	*τ*	*N*	*C*
allele	2	1	10.0
allele	2	2	15.8
genotype	1	1	14.7
genotype	1	2	23.3
genotype	2	1	27.8
genotype	2	2	44.1

Consider the example of the TGP, whose sizable *ad hoc *design effort was already mentioned above. For *N *= *τ *= 2 at the 95% level, Table 3 indicates *C *= 15.8. Assuming 1% rare allele discovery, optimal processing calls for roughly 440 samples sequenced to 3.6× each, for a project total of *R *= 1580×. Given the long-standing convention of specifying *ρ *in whole units, these results largely confirm the TGP design, though in a more precise fashion. That is, TGP has only winnowed the sample size to 400-500 per population cluster, with each sample sequenced to 4× [6,7]. The associated *P*_*v *_curve is relatively flat in 400 ≤ *σ *≤ 500, but this imprecision, coupled with a round value of *ρ*, still imposes a degree of cost liability. For instance, on the outer end, the project would expend 4·500 = 2000× in data, roughly 25% more than that required for 95% confidence. Project modifications are readily analyzed, for example, reaching alleles down to *ϕ *= 0.5% would simply require doubling the project: about 880 samples with *R *= 3160×. Analysis of genotypes is now similarly trivial.

## Conclusion

Sequence variation is often called the "currency" of genetics [4] and whole-genome sequence variation projects, enabled by continuing advances in technology, will likely become both increasingly important and routine in biomedical research. Although finding common occurrences is no longer considered to be very difficult, rare ones remain challenging because of the significantly larger amounts of data that must be gathered. Process optimization has to be considered much more carefully here. We have reported a general, though remarkably simple set of optimization principles based on analyzing the population sequencing problem. Results largely confirm the design of a special case, that of the TGP, but also permit immediate analysis of a much broader set of possible project requirements. Derivation of optimal conditions for even higher *N *and/or *τ *would be a mechanical, albeit not entirely trivial extension of the mathematics shown here, but the experimental feasibility of such designs for future projects remains unclear.

Population structure is another consideration, as rare variants are likely to be associated with particular geographic regions and their sub-populations [4]. A few issues are relevant here. First, some studies target the variation underlying specific phenotypes [21], but variant discovery projects do not place strong preference on the kinds of variation that are sought. Second, ρ* is not a function of rareness (Fig. 4), meaning that latent population-related differences in frequency will not ruin optimality. One should simply treat each desired sub-population separately, making no differential adjustments to per-sample redundancies. This strategy assures discovery of population-specific variants and, incidentally, is precisely what the TGP is following.

## Methods

### Mathematical Preliminaries

This section expands on some of the mathematical esoterica involved in establishing the theory.

#### Chain Rule

This principle enables one to find the derivative of a function that itself depends on another function [22]. In essence, it establishes a product rule for the respective derivatives. For example, if *y *= *z*^3 ^and *z *= *x*^2 ^+ 1, then *dy/dx *= *dy/dz*·*dz/dx *= *3z*^2^·2*x *= 6*x*(*x*^2 ^+ 1)^2^. Chain Rule is used in the logarithmic differentiation process described below.

#### Independently and Identically Distributed (IID)

This term means that all random variables in a collection are independent of one another, i.e. they have no mutual influences or relationships, and that each has the same probability as all the others [23]. Coin flipping is a simple example. The current flip is not influenced by past ones, nor does it influence future ones, and each flip has the same probability of showing, say, "heads". This concept is the basis of ultimately establishing the binomial nature of *P*_*v *_in Theorem 1.

#### Logarithmic Differentiation

This mathematical device employs the Chain Rule (see above) to differentiate functions whose forms render them difficult to handle using more basic rules. Proof of Theorem 3 (below) requires this treatment because the independent variable being differentiated against appears in the exponent. An illustrative example having precisely the same issue is *y *= *e*^*x*^, which is readily shown by this procedure to be its own derivative. Applying Chain Rule to the logarithmic form, ln *y *= *x*, yields *y*^-1^·*dy/dx *= 1, from which *dy/dx *= *y *= *e*^*x *^immediately follows.

#### Notation

This aspect is complicated by the fact that the theory straddles two different branches of mathematics: probability and calculus. In the former case, notation is primarily concerned with specifying configurations of events, while in the latter, Euler's convention is used to describe functional dependence on a set of independent variables. This necessitates a change in notation as we move from the probabilistic discovery model in Thms. 1 and 2 to the calculus-based optimization process in Thm. 3.

Substituting the constraint in Eq. 5, *ρ *= *R*/*σ*, into Eq. 2, we can write the *constrained *form of the coverage probability as(11)

which now depends upon *τ*, *R*, and *σ*. In turn, this expression is substituted into Eqs. 1 and 4 to obtain constrained probabilities for events *D*_*A *_and *D*_*G*_, respectively, with dependence now extending to *ϕ*, as well. From here on, let us consider these event probabilities simply as mathematical functions. For example, *f*_1, *G *_is the expression obtained by setting *τ *= 1 in Eq. 11, squaring it, and multiplying by *ϕ*, i.e. it is the constrained probability of the event *D*_*G *_originally introduced in Eq. 4. Under this notation, we can then easily represent *all *such functions universally by writing them in a form *f*_*τ*, *i *_= *f*_*τ*, *i *_(*ϕ*, *R*, *σ*), where *i *∈ {*A*, *G*}. This is the convention we follow in both Thm. 3 (above) and its proof (below).

#### Roots of a Function

Roots are values of the independent variable which cause a function to vanish, i.e. to be equal to zero. For example, *y *= *x*^2 ^- 9 can be factored as *y *= (*x *+ 3) (*x *- 3), showing that *x *= ±3 are the roots for which *y *= 0. This concept is relevant to the proof of Theorem 3 (below) because maxima within the *P*_*v *_family of functions occur at roots in *σ *of the first derivatives. Roots play a similar role in solving Eqs. 15 and 16.

### Proofs of Theorems 1 to 3

**Theorem 1**: Let *A*_*j *_and *C*_*j *_be the events, respectively, that an allele variant exists on chromosome *j *in a sample at location *x *and that *x *is spanned (covered) by at least *τ *sequence reads. The detection event is *D*_*A *_= (*A*_1 _∩ *C*_1_) ∪ (*A*_2 _∩ *C*_2_). Given the presumed IID (see "Mathematical Preliminaries") nature of alleles and coverage with respect to chromosomes, *ϕ *= *P*(*A*_1_) = *P*(*A*_2_) and *P*(*C*) = *P*(*C*_1_) = *P*(*C*_2_), from which Eq. 1 follows directly. Eq. 2 is a corollary of diploid covering theory [24]. Finally, with respect to any given sample, *D*_*A *_is a Bernoulli process: an allele is either detected, or it is not. Given uniform *ρ *for each sample and the random selection of presumably independent genomes, the process is IID. The distribution of detected variants is then binomial [23], from which Eq. 3 follows directly.

**Theorem 2**: Let *G *represent the existence of a rare genotype in a sample. Since both alleles must be discerned, the detection event is *D*_*G *_= *G *∩ *C*_1 _∩ *C*_2_. Because coverage of *x *is not a function of whether the genotype is actually present and *vice versa, G *and *C*_1 _∩ *C*_2 _are independent, whereby Eq. 4 follows directly.

**Theorem 3**: The optimization problem is cast by substituting the single-sample detection probability, *f*_*τ*, *i *_(see "Mathematical Preliminaries"), into the project-wide discovery probability, *P*_*v*_(*K *≥ *N*) in Eq. 3. Noting that *f*_*τ*, *i *_and *P*_*v *_are both functions of *σ *(among other variables), but omitting the functional notation, this process gives(12)

for the special cases of interest, *N *= 1 and *N *= 2, respectively.

Roots in *σ *of the first derivatives of these equations are a necessary condition in identifying the extrema of *P*_*v*_[22]. Their forms require us to use logarithmic differentiation. (This procedure and the concept of roots are both outlined in the "Mathematical Preliminaries" section above.) Setting the resulting derivatives equal to zero gives the corresponding characteristic equations

and

for *N *= 1 and *N *= 2, respectively. In general, *P*_*v *_≠ 1 in either case, so the conditions must instead be satisfied by the terms in square brackets. Eqs. 7 and 8 follow directly.

The fact that there is only a single, global optimum, σ*, for each case is a consequence of *P*_*v *_being a unimodal function in *σ*. In general, *P*_*v *_vanishes monotonically for *σ *>σ* because *P*(*C*) → 0, and consequently *f*_*τ*, *i *_→ 0, as *σ *is increased under finite values of *R*. The exception is *f*_1, *A*_, for which *P*_*v *_asymptotically approaches a maximum (Eq. 10).

### Solution of the General Optimization Problem

Optimal conditions are described by constants of ρ*, which can be substituted into the single-sample probability to obtain an optimized . For *N *= 1, we can then derive the following expression, valid for both alleles and genotypes, directly from Eq. 12(14)

where constants *λ*_*τ *_and *β*_*τ *_are given in Table 2. This equation describes the relationship between *ϕ *and *R *under optimal conditions when given user-specified values of *τ *and *P*_*v*, *min*_. For *N *= 2, we cannot readily obtain an explicit optimization rule from Eq. 13. Instead, we cast the relationship as a root-finding problem in *ϕ *for genotypes as(15)

and for alleles as(16)

That is, given *τ*, *R*, and *P*_*v*, *min *_the values of *ϕ *under which the process is optimal are the roots of Eqs. 15 and 16.

### Derivatives and Numerical Method

Eqs. 7 and 8 depend upon partial derivatives of *f*_*τ*, *i*_. For rare alleles and genotypes, i.e. *i *∈ {*A*, *G*}, we follow standard rules of differentiation [22] to obtain(17)

Note that an equation for *f*_1, *A *_is absent because the case of *τ *= 1 for rare alleles does not have a well-defined optimum (Eq. 10).

Eqs. 7, 8, 15, and 16 all depend upon the concept of finding the roots of an equation. (See "Mathematical Preliminaries" above.) Although none is readily factorable, they can be solved by the bisection algorithm, which is straightforward to apply, has reasonably good convergence behavior, and is extremely robust [25].

## Abbreviations

NGI: next-generation sequencing instrument; TGP: Thousand Genomes Project.

## Authors' contributions

MCW conceived and constructed the mathematical theory and wrote the paper. Both authors approved the final manuscript.
